# Zeaxanthin Dipalmitate-Enriched Emulsion Stabilized with Whey Protein Isolate-Gum Arabic Maillard Conjugate Improves Gut Microbiota and Inflammation of Colitis Mice

**DOI:** 10.3390/foods11223670

**Published:** 2022-11-16

**Authors:** Xuhui Kan, Wangting Zhou, Weiqi Xu, Zhuqing Dai, Yamei Yan, Jia Mi, Yi Sun, Xiaoxiong Zeng, Youlong Cao, Lu Lu

**Affiliations:** 1 College of Food Science and Technology, Nanjing Agricultural University, Nanjing 210095, China; 2 Institute of Agro-Product Processing, Jiangsu Academy of Agricultural Sciences, Nanjing 210014, China; 3 Institute of Wolfberry Engineering and Technology, Ningxia Academy of Agriculture and Forestry Sciences, Yinchuan 750004, China

**Keywords:** *Lycium barbarum*, zeaxanthin dipalmitate, protein-polysaccharide conjugate, gut microbiota, colitis

## Abstract

In the present study, protein-polysaccharide Maillard conjugates were used as novel emulsifiers and bioactive carriers. Effects and potential mechanisms of zeaxanthin dipalmitate (ZD)-enriched emulsion stabilized with whey protein isolate (WPI)-gum Arabic (GA) conjugate (WPI-GA-ZD) and ZD-free emulsion (WPI-GA) on gut microbiota and inflammation were investigated using a model of dextran sulfate sodium (DSS)-induced colitis in mice. As a result, supplementation with WPI-GA and WPI-GA-ZD improved the serum physiological and biochemical indicators, decreased the expression of pro-inflammatory cytokines and related mRNA, as well as increased the tight junction proteins to a certain extent. 16S rDNA sequencing analyses showed that supplementation with WPI-GA and WPI-GA-ZD presented differential modulation of gut microbiota and played regulatory roles in different metabolic pathways to promote health. Compared with WPI-GA, the relative abundances of *Akkermansia*, *Lactobacillus* and *Clostridium_IV* genera were enriched by the intervention of WPI-GA-ZD. Overall, the designed carotenoid-enriched emulsion stabilized with protein-polysaccharide conjugates showed potential roles in promoting health.

## 1. Introduction

Inflammatory bowel disease (IBD) is a chronic, relapsing, and unpredictable inflammatory disorder that typically includes Crohn’s disease (CD) and ulcerative colitis (UC). The pathogenesis of IBD is complex and associated with multiple factors. In addition to the influence of genetic factors, environmental factors, especially diet, can significantly influence the development of IBD [1,2]. Several explanations have been advanced from different perspectives on how diet affects the development of IBD. On the one hand, diet could alter the microbial community composition, metabolism, and function. These changes exert important effects on the regulation of intestinal homeostasis [1]. On the other hand, dietary composition can regulate mucosal barrier function and may directly influence immune function [3]. Recently developed complex dietary interventions, such as the exclusion diet for CD and the specific carbohydrate diet, have been shown to be effective in reducing inflammatory conditions in IBD patients [4].

Carotenoids are fat-soluble natural pigments with enhancing immune function, antioxidant and anti-inflammatory properties, vision protection, inhibition of certain cancers and prevention of cardiovascular diseases, which can be ingested in esterified or free form. Diet is the sole source of carotenoids for humans and animals. A large proportion of dietary carotenoids would reach the colon and interact with the gut microbiota due to their relatively poor bioavailability [5]. Some free carotenoids (such as lutein, zeaxanthin, astaxanthin and lycopene) have shown the potential benefits of improving colitis [6,7,8]. Zeaxanthin dipalmitate (ZD) is an esterified form of zeaxanthin, as well as the major carotenoid in fully ripe fruits of *Lycium barbarum* L. [9]. Despite carotenoids have been recognized as natural pigments with important health-promoting effects, most studies are focused on free carotenoids but not carotenoid esters. Thus, knowledge about the function of ZD and its effects on health remains unclear.

Emulsion-based delivery systems are good delivery approaches for carotenoids and other hydrophobic phytochemicals. Emulsifier plays an important role in emulsion formulations, which could stabilize the emulsion and have a major impact on the functional property of the final product [10]. As a major addition to the human diet, the role of dietary emulsifiers in the development of IBD is often overlooked. Recently, a range of studies showed a positive correlation between IBD and the intake of certain types of emulsifiers, for example, polysorbate-60 (P60) and P80, which belong to surfactants, and some polysaccharides such as carrageenan (CGN) and carboxymethylcellulose (CMC) [4,11]. These dietary emulsifiers might promote inflammatory disorders by directly impacting the gut microbiota. With the increasing demand from consumers for natural and healthy food products, the utilization of mixtures of biopolymer-based emulsifiers would be the focus of the food industry. Novel emulsifiers resulting from the Maillard conjugation between food proteins and polysaccharides received widespread concern owing to their improved functional attributes [10,12]. In a previous in vitro study, we successfully prepared the protein-polysaccharide Maillard conjugates and confirmed that emulsion stabilized with the conjugates could improve gut microbiota by enriching potential probiotics *Bacteroides ovatus* and *Bifidobacterium longum*. Besides, this delivery system might promote health by enriching the pathways of carbohydrate metabolism and bile acid biosynthesis [13]. However, at present, the impact of Maillard conjugates as dietary emulsifiers on intestinal inflammation and gut microbiota in vivo has not been fully elucidated. Hence, further investigation is required to ensure that the novel emulsifiers would not cause adverse effects on the gut microbiota or host.

Diet usually contains various bioactive ingredients, and the exclusion of other ingredients from complex diets presents a challenge. As over-the-counter food supplements, commercial carotenoid products usually contain numerous additives due to their poor solubility and low bioavailability. Previous studies investigated the health-promoting effects of a single ingredient but ignored the possible synergistic effects among these bioactive ingredients. Therefore, a model of dextran sulfate sodium (DSS)-induced colitis in mice was used in the current study to investigate the effects of emulsion stabilized with the protein-polysaccharide conjugates on gut microbiota in vivo and whether ZD would act synergistically with protein-polysaccharide conjugates to show better effects in protecting the intestinal barrier, inhibiting the inflammatory response, and reshaping the gut microbiota of mice with colitis. The objective of this study was to provide some new insights into the health effects of carotenoid esters and protein-polysaccharide conjugates in the emulsion system.

## 2. Materials and Methods

### 2.1. ZD Purification and Emulsions Preparation

ZD with a purity ≥93% was prepared from the fully ripe fruits of *L. barbarum* by using high-speed countercurrent chromatography based on our previous method [9]. Whey protein isolate (WPI, protein content >90%)-gum Arabic (GA, weight-average molecular weight 250 kDa) Maillard conjugates were prepared as the emulsifier by the dry-heating method [13]. In short, WPI-GA Maillard conjugates with a degree of grafting of 34.9 ± 1.7% were obtained by incubating the mixtures of WPI and GA for 3 days under suitable conditions. The emulsifier was firstly dissolved in an aqueous solution (60%, *w*/*w*) and then homogenized with olive oil (40%, *w*/*w*) with or without ZD at 14,000 rpm for 5 min to afford the coarse emulsion. The coarse emulsion was then immediately processed to homogenized under ultra-high pressure for 10 cycles at 13,500 psi. The resulting emulsion (containing 3% WPI-GA conjugates, *w*/*w*) was stored at 4 °C and used within 8 days. For convenience, we used WPI-GA to represent ZD-free emulsion and WPI-GA-ZD to represent ZD-enriched emulsion.

### 2.2. Characterization of Emulsions

The microstructure of emulsions was characterized by a TCS SP8 CLSM (Leica Microsystems, Wetzlar, Germany). Nile Red (1.0 mg/mL, dissolved in the olive oil) and Nile Blue A (1.0 mg/mL, dissolved in the aqueous phase) were used as the dyes to stain the oil phase and proteins, respectively. The particle size distribution and ξ-potential of emulsions were determined by a laser diffraction particle size analyzer (Mastersizer 3000, Malvern Instruments Ltd., Worcestershire, UK) and a Zetasizer (Nano ZS90, Malvern Instruments Ltd.), respectively. Detailed results of characterization are shown in the supplementary material (Figure S1).

### 2.3. Mice Treatments

Six-week-old specific-pathogen-free (SPF) male C57BL/6J mice were purchased from Charles River Laboratories (Beijing, China). The mice have housed four individuals per cage under standard laboratory conditions with ad libitum access to standard chow and sterile drinking water. The protocol was approved by the Ethics Committee (Name: Animal Ethics Committee of Nanjing Agricultural University; Approval Code: NJAU.No20210310011; Approval Date: 20210310), and the experiments were conducted in accordance with the National Guidelines for Experimental Animal Welfare. After acclimation for 7 days, the mice were randomly distributed into groups (four groups in total, eight individuals per group): normal control (NC) group, DSS-induced colitis model (DSS) group, DSS-induced colitis + WPI-GA (EMU) group, and DSS-induced colitis + WPI-GA-ZD (EZD) group. Mice were induced to develop colitis by administering 1.5% (*w*/*v*) DSS with molecular weights of 36,000–50,000 Da in sterile drinking water for 8 days in the DSS, EMU and EZD groups, whereas the mice in the NC group were given DSS-free sterile drinking water. The WPI-GA and WPI-GA-ZD (100 mg/kg/d) were administered to the EMU and EZD groups by intragastric gavage (0.2 mL per day) from day 9 to day 16, respectively, and the mice in other groups received the same volume of an oil-water mixture in the absence of WPI-GA conjugate with the same oil-water mass ratio as the prepared emulsion. The main clinical symptoms of mice, including weight of the body and solid fecal, food consumption and disease activity index (DAI), were monitored daily during the experiments. The DAI score was the combined score of body weight loss, stool consistency and rectal bleeding and was calculated according to the following scoring rules: weight loss (0, <1.5%; 1, 1.5~5%; 2, 5~10%; 3, 10~15%; 4, >15%), stool consistency (0, normal; 2, loose; 4, diarrhea), and degree of bleeding (0, absent; 2, slight bleeding; 4, gross bleeding) [14]. After the sample intervention was completed (day 17), all mice were sacrificed by cervical dislocation. Excised the entire colon and measured its length. A segment of the colon for histological observation and immunofluorescence staining was collected, and the remaining tissue was used for molecular analysis. In addition, the cecal contents, serum, and feces samples were collected and stored at −80 °C for the follow-up experiments.

### 2.4. Determinations of Serum Biochemical Parameters

Based on the manufacturer’s instructions, levels of biochemical parameters, including tumor necrosis factor -alpha (TNF-α), interferon -gamma (IFN-γ), interleukin-6 (IL-6), monocyte chemoattractant protein-1 (MCP-1), prostaglandin E2 (PGE2) and lipopolysaccharides (LPS) in serum were determined using commercial kits (Yifeixue Bio. Tech., Nanjing, China; Nanjing Jiancheng Bioengineering Institute, Nanjing, China).

### 2.5. Histological Analysis

The fresh colon tissue was collected and fixed in 10% (*w*/*v*) formalin solution for more than 24 h. Paraffin-embedded sections were stained with hematoxylin and eosin (H&E) staining and alcian blue/periodic acid-Schiff staining (AB-PAS), respectively. Observation of colon tissue’s pathological conditions (including crypt damage, the extent of inflammatory infiltration, edema, ulceration, and the number of goblet cells) under a light microscope and the scanning images were recorded to characterize histological changes. The colons were scored according to the previous report that graded the extent of inflammatory infiltrate, ulceration, crypt damage and the presence or absence of edema [15]. The number of goblet cells was counted using ImageJ open-source software (version number 1.48, National Institutes of Health, Bethesda, USA).

### 2.6. Immunofluorescence Staining

The paraffin sections of the colon were stained with immunofluorescence using antibodies targeting the tight junction proteins according to the standard protocol. Briefly, the primary antibody was incubated with the fixed colon tissue at 4 °C overnight and then rinsed with PBS, and the resulting tissue was incubated with FITC-labeled secondary antibody at room temperature for 1 h under dark conditions. After counterstaining with 4,6-diamidino-2-phenylindole (DAPI), an UltraView ERS confocal laser scanning microscopy was used to capture the fluorescent images of samples. Relative fluorescence intensity was measured using ImageJ 1.48 open-source software.

### 2.7. Determination of Short-Chain Fatty Acids (SCFAs)

An appropriate amount of cecal contents were mixed with water thoroughly to release the SCFAs, then the mixture was centrifuged at 5000 rpm for 10 min. The resulting supernatants were collected and mixed with a 2-ethylbutyric acid solution (25 mM) used as an internal standard in equal volume for SCFAs analysis. According to the reported method, the contents of SCFAs in the cecal contents were determined using gas chromatography [16].

### 2.8. RNA Extraction and Quantitative Real-Time PCR (RT-qPCR) Analysis

According to the manufacturer’s instructions, extracted total RNA from the colon tissue by using a commercial kit (Vazyme Biotech Co., Ltd., Nanjing, China). Complementary DNA (cDNA) was synthesized via RNA reverse-transcription using the HiScrip III RT SuperMix (Vazyme Biotech Co., Ltd.). The amplification and detection of RT-qPCR were performed with ChamQ Universal SYBR qPCR Master Mix (Vazyme Biotech Co., Ltd., Nanjing, China). The relative mRNA expression levels of target genes were calculated according to the 2^–ΔΔCT^ quantification method, and glyceraldehyde-3-phosphate dehydrogenase (GAPDH) was selected as the housekeeping gene. All primer sequences used in RT-qPCR assays are shown in Table S1.

### 2.9. 16S rDNA Gene High-Throughput Sequencing

The total genomic DNA from the feces sample was extracted, and the quality of the DNA was checked using agarose gel electrophoresis. DNA sequences were amplified with high-fidelity PCR using specific primers for the V3-V4 hypervariable regions of the 16S rDNA gene, and high-throughput sequencing was conducted at Genesky Biotechnologies Inc., Shanghai, China. All results were based on the sequenced reads and unique amplicon sequence variants (ASVs).

### 2.10. Statistical Analysis

Experimental data are expressed as mean ± standard deviation (SD). Differences between groups were analyzed according to a one-way analysis of variance (ANOVA) with a Duncan multiple-range test and a Wilcoxon rank-sum test using IBM SPSS Statistics 25 (SPSS Inc., Chicago, IL, USA). The KEGG (Kyoto Encyclopedia of Genes and Genomes) pathway differences and bacterial gene function predictions were determined by Phylogenetic Investigation of Communities by Reconstruction of Unobserved States (PICRUSt).

## 3. Results

### 3.1. WPI-GA and WPI-GA-ZD Relieved the Symptoms of DSS-Induced Colitis in Mice

**Figure 1:**
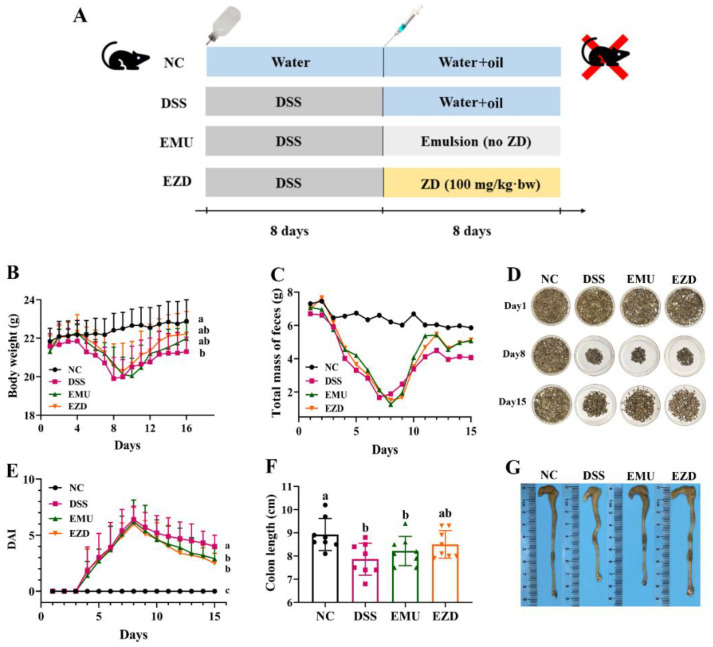
Effects of WPI-GA and WPI-GA-ZD on symptoms changes of DSS-induced colitis. (**A**) Experimental schedule, (**B**) Body weight, (**C**) Total mass of feces, (**D**) Images of solid feces, (**E**) DAI score, (**F**) Colon length, (**G**) Representative images of the colon. Data are expressed as mean ± SD (*n* = 8), and different letters represent significant differences (*p* < 0.05).

In comparison with the NC group, the body weight and solid fecal weight of mice decreased apparently during the modeling period with DSS, whereas the DAI score increased rapidly (Figure 1B,C,E). The solid feces of the mice in the DSS group not only decreased in weight but also had a darker color compared with the NC group (Figure 1D). Mice treated with the WPI-GA or WPI-GA-ZD showed an improvement in these pathological features and DAI scores. On the last day of treatment, the mice in EMU and EZD groups showed a better recovery trend in body weight and solid fecal weight, and a significantly lower DAI score (*p* < 0.05) was observed. The colon length of mice treated with DSS was significantly shortened (*p* < 0.05, Figure 1F,G). The order of colon length in different groups was as follows: NC group > EZD group > EMU group > DSS group.

### 3.2. WPI-GA and WPI-GA-ZD Decreased the Levels of Proinflammatory Cytokines and Endotoxins

The serum levels of pro-inflammatory cytokines and endotoxin in the DSS group, including TNF-α, IFN-γ, IL-6, LPS, MCP-1 and PGE2, were significantly increased (*p* < 0.05, Figure S2) in comparison with the NC group. Both WPI-GA and WPI-GA-ZD could reverse this inflammatory-response phenotype to some extent. Supplementation with WPI-GA led to an obvious decrease in IL-6, LPS and PGE2, whereas WPI-GA-ZD decreased the levels of LPS, MCP-1 and PGE2 significantly (*p* < 0.05). Compared with the DSS group, the levels of TNF-α and IFN-γ in the EMU and EZD groups were also lower, but no significant difference was observed.

### 3.3. WPI-GA and WPI-GA-ZD Reduced Histological Damage of Colon

**Figure 2:**
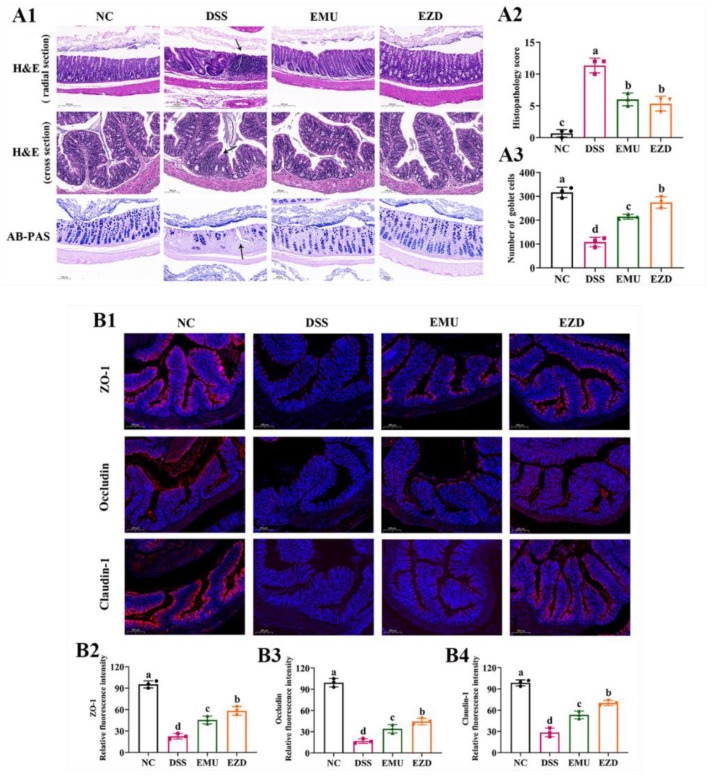
(**A**) Effects of WPI-GA and WPI-GA-ZD on histological damage of colon induced by DSS. (**A1**) Representative histological sections of colonic tissue stained with H&E and AB-PAS. Black arrows pointed to the features we want to highlight in the DSS group: large-scale ulceration in the mucosa layer, intense infiltration of abundant neutrophils and monocytes, distorted crypts, and mass loss of goblet cells, (**A2**) Histopathological score, (**A3**) Number of goblet cells. Data are expressed as mean ± SD (*n* = 3). Different letters represent significant differences among different groups (*p* < 0.05). (**B**) Effects of WPI-GA and WPI-GA-ZD on the levels of tight junction proteins. (**B1**) Immunofluorescence staining analysis representative images of ZO-1, occludin, and claudin-1 for each group, (**B2**) Relative fluorescence intensity of ZO-1, (**B3**) Relative fluorescence intensity of occluding, (**B4**) Relative fluorescence intensity of claudin-1. Data are expressed as mean ± SD (*n* = 3), and different letters represent significant differences among different groups (*p* < 0.05).

The colon tissues stained with H&E and AB-PAS were histologically examined to observe the DSS-induced pathological damage (Figure 2A1). Representative colon tissue sections of the NC group showed intact crypt, stroma, surface epithelia, and submucosa. As for the colon tissue of the DSS group, large-scale ulceration in the mucosa layer, intense infiltration of abundant neutrophils and monocytes, notable distortion of crypts and mass loss of goblet cells were observed. Compared with the DSS group, the representative histological colon sections of the EMU and EZD groups presented improved histological features, including relatively well-preserved crypt structures, fewer infiltrations of inflammatory cells and more goblet cells, resulting in a significant decrease in the histological scores (Figure 2A2,A3).

### 3.4. WPI-GA and WPI-GA-ZD Improved the Levels of Tight Junction Proteins

Zonula occludins (ZO), occludin, and claudin family proteins are important tight junction proteins belonging to the epithelial barrier. The epithelial tight junction proteins and intestinal mucus work together to maintain colonic barrier integrity [17]. As the prime pathological feature of colitis, the damage of the physical colonic barrier is often accompanied by the decreased expression of tight junction proteins. The beneficial effects of WPI-GA and WPI-GA-ZD on ZO-1, occludin and claudin-1 were confirmed by the immunofluorescence staining analysis. Compared with the NC group, three tight junction proteins located on the epithelial cell membrane almost disappeared in the DSS group (Figure 2B1), and the corresponding relative fluorescence intensity was significantly reduced (*p* < 0.05, Figure 2B2–B4). After the intervention of WPI-GA or WPI-GA-ZD, the expression of tight junction proteins was enhanced, which might improve the integrity of the intestinal barrier.

### 3.5. WPI-GA-ZD Increased the SCFAs Contents

The contents of acetic, butyric, valeric and total acids in cecal contents were significantly decreased after DSS treatment (Figure S3). Supplementation of WPI-GA significantly increased the contents of *i*-butyric and *i*-valeric acids, whereas WPI-GA-ZD significantly increased the contents of acetic, *i*-butyric, *i*-valeric and total acids. The contents of *n*-butyric and *n*-valeric acids also showed a trend of recovery after supplementing WPI-GA and WPI-GA-ZD (not significantly). In comparison with WPI-GA, WPI-GA-ZD showed a better recovery effect on SCFAs, especially for acetic acid.

### 3.6. WPI-GA and WPI-GA-ZD Regulated the mRNA Expression of Recognition Receptors and Cytokines

Various recognition receptors and cytokines that related to colitis in colon tissues were measured at the mRNA level. Consistent with the results of immunofluorescence staining, the mRNA expression of key markers related to tight junctions, such as ZO-1, occludin and claudin-1, decreased significantly (*p* < 0.05) in the DSS group. Supplementing WPI-GA and WPI-GA-ZD increased the mRNA expression of tight junctions, but no significant difference in ZO-1 and occludin expression was observed in the EMU group (Figure S4A–C). The mRNA expression of pro-inflammatory cytokines (IL-1β and IFN-γ) and the inducible nitrogen oxide synthase (iNOS) stimulated by pro-inflammatory cytokines increased significantly (*p* < 0.05) in the DSS group. WPI-GA-ZD significantly reduced (*p* < 0.05) all these mRNA expressions, whereas WPI-GA did not reduce the expression of IFN-γ effectively (Figure S4D–F). GPR41, GPR43 and GPR109A are the main receptors of SCFAs involved in the anti-inflammatory mechanisms of SCFAs. In comparison with the NC group, DSS significantly decreased (*p* < 0.05) the mRNA expression of GPR41, GPR43 and GPR109A. Supplementation with WPI-GA-ZD significantly increased (*p* < 0.05) the expression of these receptors, whereas no significant increased trend of GPR43 was observed in the EMU group (Figure S4G–I).

### 3.7. WPI-GA and WPI-GA-ZD Regulated the Gut Microbiota Structure

**Figure 3:**
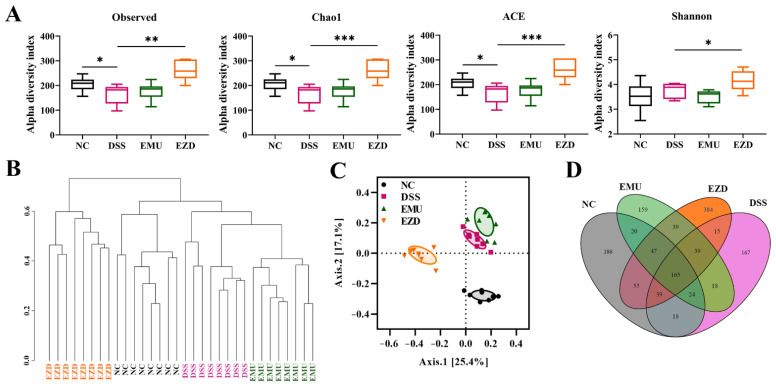
Effects of WPI-GA and WPI-GA-ZD on gut microbiota. (**A**) Alpha diversity analysis, (**B**) Samples cluster tree, (**C**) Principal coordinate analysis, (**D**) Venn diagram. Data are expressed as mean ± SD (*n* = 8), and statistical significance (* *p* < 0.05, ** *p* < 0.01, and *** *p* < 0.001) was determined using the Wilcoxon rank-sum test.

According to the alpha diversity analysis (Figure 3A), compared with the NC group, the community richness (Observed, Chao1 and ACE indexes) of the DSS group decreased significantly (*p* < 0.05). Supplementation with WPI-GA-ZD significantly improved the community richness and diversity (Shannon index) of gut microbiota in the EZD group (*p* < 0.05), but no significant difference was observed in the EMU group (*p* > 0.05). Samples cluster tree and principal coordinate analysis (PCoA) were performed to analyze the beta diversity. As the cluster tree showed, the microbial compositions of the NC, DSS, EMU and EZD groups were distinct as they were located on different branches (Figure 3B). Also, the difference in gut microbiota caused by different treatments was observed in the PCoA (Figure 3C). Especially compared with the EMU group, the sample point distribution in the EZD group was farther away from the sample points in the DSS group. As the Venn diagram results (Figure 3D) showed, there were 186, 167, 159 and 384 unique ASVs in the NC, DSS, EMU and EZD groups, respectively. These results indicated that WPI-GA and WPI-GA-ZD had different modulating effects on the gut microbiota.

**Figure 4:**
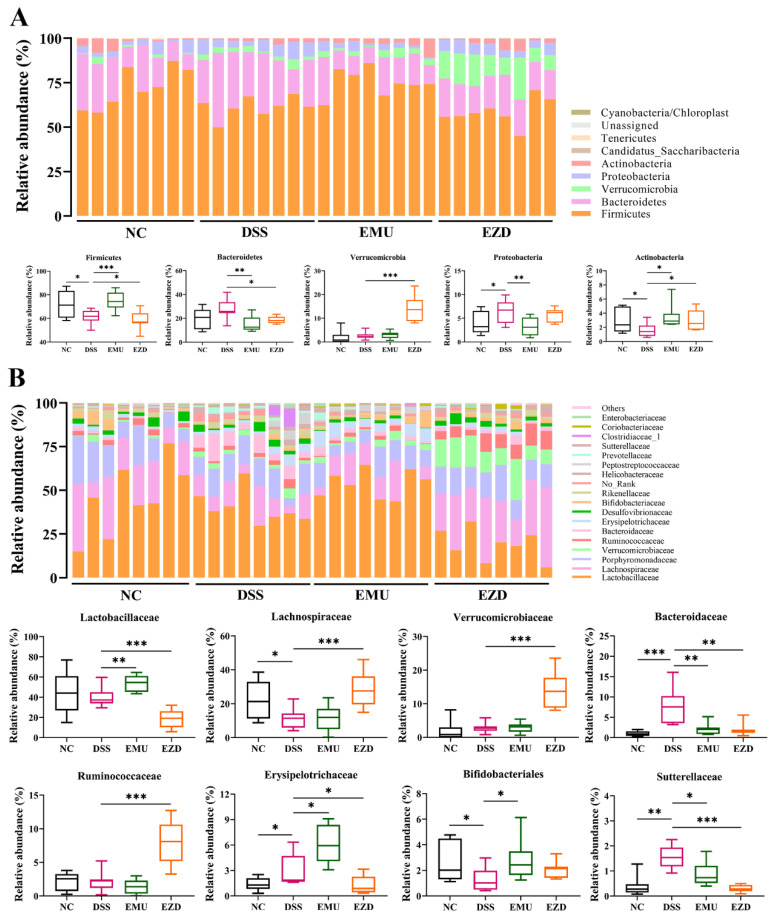
Effects of WPI-GA and WPI-GA-ZD on gut microbiota community changes at (**A**) the phylum and (**B**) the family level. Relative abundance (%) is expressed as mean ± SD (*n* = 8). * *p* < 0.05, ** *p* < 0.01, and *** *p* < 0.001.

Changes in the gut microbiota community were analyzed at the phylum and family levels. Firmicutes, Bacteroidetes, Verrucomicrobia, Actinobacteria and Proteobacteria were the main microorganisms at the phylum level (Figure 4A). In comparison with the NC group, DSS treatment significantly decreased the relative abundances of Firmicutes and Actinobacteria but increased the relative abundance of Proteobacteria. Supplementing WPI-GA increased the relative abundance of Firmicutes and Actinobacteria but decreased the relative abundance of Bacteroidetes, whereas WPI-GA-ZD dramatically increased the relative abundance of Verrucomicrobia. From the family level of bacterial composition (Figure 4B), Lachnospiraceae was much absent after the DSS treatment, but the relative abundances of Bacteroidaceae, Sutterellaceae and Erysipelotrichaceae were remarkably increased. Both WPI-GA and WPI-GA-ZD supplementation could significantly decrease the relative abundance of Bacteroidaceae. Differently, the relative abundances of Lactobacillaceae and Erysipelotrichaceae were increased in the EMU group, whereas the intervention with WPI-GA-ZD significantly increased the relative abundances of Lachnospiracea, Verrucomicrobiaceae and Ruminococcaceae.

**Figure 5:**
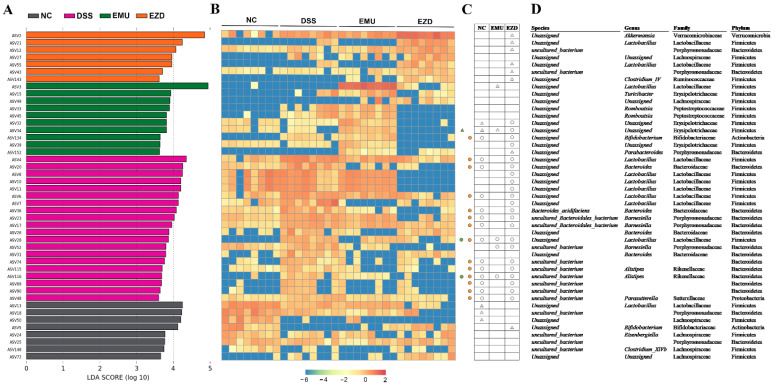
(**A**) Linear discriminant analysis (LDA) of gut microbiota based on ASVs (LDA score > 3.6), (**B**) Heatmap shows the relative abundance of ASVs that was significantly altered based on LDA in different groups, (**C**) Compared with the DSS group, the circles (○) and triangles (△) represent the significantly less and more relative abundances of ASVs, respectively. The colorful circles and triangles represent that the changes of ASVs in the EMU or EZD group had the same trend as the changes in the NC group, (**D**) Corresponding bacterial taxa information of different treatments.

The effects of WPI-GA and WPI-GA-ZD supplementation on the composition of gut microbiota were further evaluated using linear discriminant analysis (LDA) effect size (LEfSe) analysis. ASVs with a relative abundance of more than 1% in all groups were analyzed to identify significantly altered intestinal gut microbiota. As a result, 45 dominant ASVs (LDA score > 3.6) from the four groups were displayed together with the heatmap (Figure 5A,B). In comparison with the DSS group, the relative abundances of *Lactobacillus*, Porphyromonadaceae and Lachnospiraceae were enriched in the NC group. WPI-GA and WPI-GA-ZD showed different effects on the gut microbiota composition. Three distinct ASVs (Erysipelotrichaceae, *Lactobacillus* and *Alistipes*) were significantly reversed by WPI-GA, whereas 14 ASVs, including *Bifidobacterium*, *Lactobacillus*, *Bacteroides*, *Barnesiella*, Bacteroidetes, *Alistipes* and *Parasutterella* were significantly reversed by WPI-GA-ZD. Notably, the relative abundances of 8 ASVs were remarkably increased after the WPI-GA-ZD treatment. Among them, 1 ASV (ASV 2) belonged to the genus of *Akkermansia*, 2 ASVs (ASV21 and ASV55) belonged to the genus of *Lactobacillus*, 3 ASVs (ASV12, ASV43 and ASV152) belonged to the family of Porphyromonadaceae, 1 ASV (ASV143) belonged to the genus of *Clostridium_IV* and 1 ASV (ASV5) belonged to the genus of *Bifidobacterium*.

### 3.8. Function Prediction of Gut Microbiota Genes

**Figure 6:**
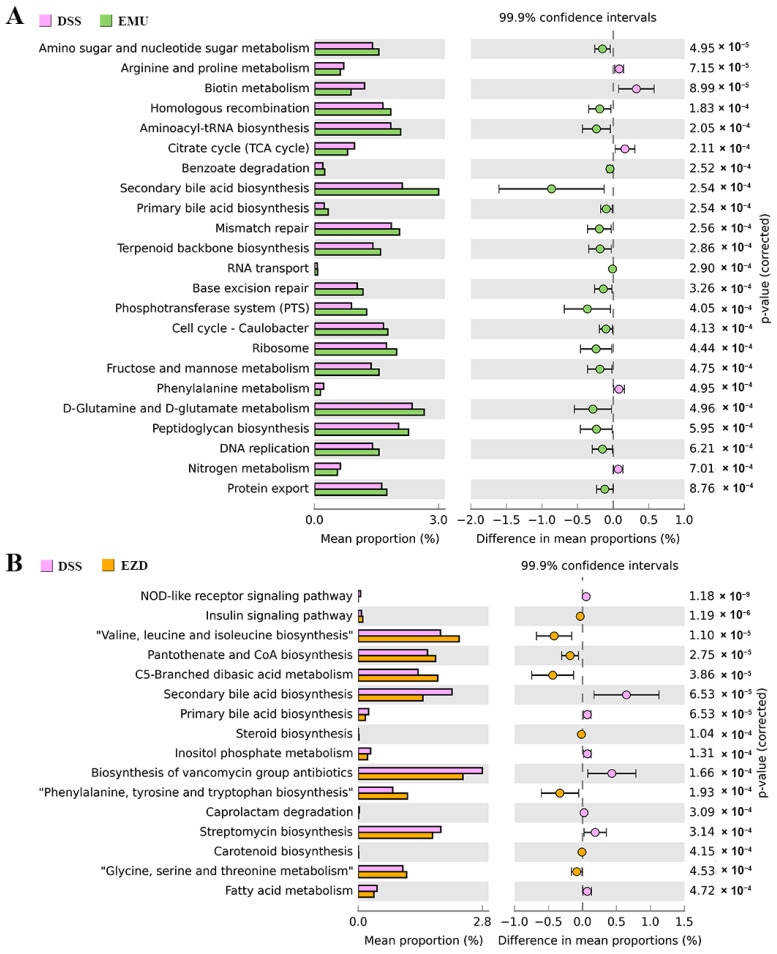
KEGG functional prediction of gut microbiota with a significant difference: (**A**) DSS group and EMU group, (**B**) DSS group and EZD group.

The functional difference between different treatment groups predicted by PICRUSt is shown in Figure 6. For the EMU and EZD groups, 23 (18 increased, 5 decreased) and 16 (8 increased, 8 decreased) functional modules were significantly altered (*p* < 0.001) in comparison with the DSS group, respectively. WPI-GA mainly promoted the metabolism of amino sugar and nucleotide sugar, D-glutamine and D-glutamate, fructose and mannose, as well as biosynthesis of aminoacyl-tRNA, secondary bile acid, terpenoid backbone and peptidoglycan. As for the EZD group, supplementation with WPI-GA-ZD could reduce nucleotide-binding oligomerization domain (NOD)-like receptor signaling pathway, whereas it enhances the insulin signaling pathway. The changed metabolic pathways of gut microbiota mainly involved lipid metabolism (“fatty acid metabolism”, “steroid biosynthesis”, “primary bile acid biosynthesis” and “secondary bile acid biosynthesis”), nucleotide metabolism (“valine, leucine and isoleucine biosynthesis”, “glycine, serine and threonine metabolism” and “phenylalanine, tyrosine and tryptophan biosynthesis”), and metabolism of terpenoids and polyketides (“biosynthesis of vancomycin group antibiotics” and “carotenoid biosynthesis”).

## 4. Discussion

Hydrophobic bioactive components such as carotenoids are often delivered through the emulsion-based system to improve their bioavailability. The impact of bioactive compounds from diet on gut inflammation is generally investigated individually. However, similar to processed food, the emulsion contains a variety of dietary components that may show additive or synergistic effects. Previous studies have elucidated that some dietary emulsifiers, such as P80, CMC and CGN, can induce colitis [18]. In addition, compared with individual proteins or polysaccharides, the functional or biological properties of the protein-polysaccharide conjugates produced by the Maillard reaction might be changed. Therefore, the role of emulsifiers should be considered when designing an emulsion-based carotenoid delivery system for functional foods. In this study, the effects of ZD from the fully ripe fruits of *L. barbarum* and an emulsifier formed by the Maillard conjugation on DSS-induced colitis mice were evaluated. It was found that supplementation with WPI-GA and WPI-GA-ZD showed positive effects on relieving the symptoms of DSS-induced colitis and presented different regulatory effects on the gut microbiota.

Some typical colitis clinical presentations (weight loss, diarrhea, hematochezia, solid fecal weight loss, etc.) and pathological changes of colon tissue (colon shortening, inflammatory infiltration, crypt damage, ulceration, edema, decreased number of goblet cells, etc.) were observed in the DSS-induced colitis mice. These results are consistent with previous research conclusions [19,20]. Improved pathological features, DAI scores, and histological features of mice in the EMU and EZD groups indicated that WPI-GA and WPI-GA-ZD both showed alleviation effects on IBD.

The DSS-treated mice exhibited increased production of various pro-inflammatory cytokines in the serum, as well as an increased mRNA expression of pro-inflammatory cytokines in the colon tissue. These pro-inflammatory cytokines, such as TNF-α and IL-6, are closely related to the pathogenesis of IBD. In the current study, the administration of WPI-GA or WPI-GA-ZD decreased the levels of pro-inflammatory cytokines and endotoxins, demonstrating that the emulsifier and ZD used in this study would not induce inflammation.

The dysregulation of intestinal barrier function is related to the occurrence and development of IBD [21,22]. Epithelial cells are linked together by a highly organized apical junction complex (including tight junctions), which can form a barrier to separate the complex luminal milieu from the host. Inflammatory mediators and cytokines produced from host inflammatory reactions might cause aberrant expression of tight junctions, thereby increasing intestinal permeability and inducing immune dysfunction [22,23]. Consistent with previous research, the expression of these tight junction proteins decreased in the mice of the DSS group. WPI-GA and WPI-GA-ZD could improve the distribution of tight junction proteins and enhance the expression of ZO-1, occludin and claudin-1. Interestingly, the addition of ZD exerted better effects on maintaining the expression of tight junction proteins, thus attenuating DSS-induced gut barrier integrity damage.

As beneficial fermentation products in the gut, SCFAs played roles in inhibiting intestinal inflammation, maintaining the intestinal barrier, and modulating gut motility through different mechanisms [24,25]. SCFAs-producing bacteria are often found to be reduced in the mucosa and feces of IBD patients. Thus a reduction of SCFAs level is often observed [26]. In our study, low contents of acetate, butyrate and valerate were detected in the DSS-treated mice. Indigestible carbohydrates and proteins in the diet are known to be metabolized by microbiota in the cecum and colon to produce SCFAs [27]. Protein fermentation mostly gives rise to branched-chain fatty acids derived from valine, isoleucine, and leucine [28]. The mice fed with WPI-GA and WPI-GA-ZD were characterized by a significant increase in the cecal concentrations of branched-chain fatty acids of *i*-butyric and *i*-valeric acids. Different from the EMU group, the addition of ZD to the emulsion significantly increased the acetic acid level. Previous studies showed that other carotenoids, such as lycopene, could significantly increase the level of acetic acid in DSS-induced colitis mice [8]. In addition, SCFAs might activate anti-inflammatory signaling cascades by signaling through cell surface G-protein coupled receptors (GPCRs), such as GPR41, GPR43 and GPR109A [26]. For GPR43, the most potent activators are acetate and propionate. GPR41 is activated in the affinity order of propionate, butyrate and acetate, whereas GPR109A is mainly activated by butyrate [1]. In the current study, WPI-GA significantly up-regulated the expression of GPR41 and GPR109A but showed no significant effect on GPR43. This is consistent with the results that the contents of acetic and propionic acids were not significantly increased by WPI-GA. SCFAs also played an anti-inflammatory role in intestinal mucosa by histone deacetylases (HDACs) inhibition [26]. Previous research has shown that the use of HDAC inhibitors could inhibit colonic pro-inflammatory cytokines in experimental murine colitis [29]. Similarly, decreased mRNA expression of iNOS, IL-1β and IFN-γ was observed with the increase of SCFAs contents in the EMU and EZD groups. Of note, compared with WPI-GA, WPI-GA-ZD was more effective in increasing the SCFAs contents and inhibiting the mRNA expression of colonic pro-inflammatory cytokines, indicating a possible “cooperative role” between emulsifier and carotenoid in relieving inflammation.

Accumulating evidence indicates that changes in the composition and metabolism of gut microbiota are related to IBD pathogenesis and the regulation of intestinal homeostasis [1]. In our current research, the relative abundances of Proteobacteria and Bacteroidetes in DSS-induced colitis mice increased, whereas the relative abundance of Firmicutes decreased. Compared with healthy individuals, the most common changes observed in IBD patients were the reduction in the diversity of gut microbiota, the lower abundance of Firmicutes, and the higher abundance of Proteobacteria [30]. Dietary intake could largely shape gut microbiota of the hosts [31]. The intake of emulsion with or without ZD caused different changes in gut microbiota composition. From the analysis of the family level, WPI-GA mainly increased the relative abundances of Lactobacillaceae and Bifidobacteriaceae. The emulsion used in the EMU group mainly contained animal protein (WPI), polysaccharides (GA), and fats (olive oil). Consumption of whey protein extract, non-digestible carbohydrates and monounsaturated fatty acid-rich oil has been proven to increase the abundance of *Lactobacillus*, which belongs to Lactobacillaceae [32,33]. In vitro experiments also showed that emulsion with WPI-GA conjugates as emulsifiers could increase the relative abundance of Bifidobacteriaceae [13]. WPI-GA-ZD consumption significantly increased the relative abundances of Lachnospiraceae, Verrucomicrobiaceae and Ruminococcaceae. The relative abundance of the Verrucomicrobiaceae family, to which the *Akkermansia* genus is subordinated, has been reported to be inversely correlated with the inflammation activity in individuals with IBD [34]. Lachnospiraceae and Ruminococcaceae are the main butyrate producers residing in the intestinal microbiota [26,30]. Generally, microbial dysbiosis in IBD patients is related to the reduction of SCFAs/butyrate-producing bacteria, and most of these bacteria belong to the Lachnospiraceae and Ruminococcaceae families [26].

According to the results of LEfSe analysis at the ASV level, each group exhibited its own dominant bacteria. Notably, *Lactobacillus* (ASV3) and *Akkermansia* (ASV2) were the dominant ASVs in the EMU and EZD groups, respectively. *Lactobacillus* strains are one of the important components of the human and animal gut microbiota, which have been proven to promote host health and exert anti-inflammatory effects in UC remission [35,36]. Previous studies showed that *Lactobacillus* strains could alleviate colonic inflammation and injury by inhibiting cytokine-induced apoptosis, reducing the expression of inflammatory cytokines, maintaining epithelial barrier integrity, and promoting the secretion of SCFAs [35,37]. *Akkermansia* is a potential probiotic agent with demonstrated ability to regulate intestinal barrier function, metabolic functions, and inflammation of the host. As a specifically efficient mucin-degrading bacterium, *Akkermansia* can produce acetate and propionate using intestinal mucins, thus contributing to intestinal health and glucose homeostasis. Besides, this bacterium could improve intestinal integrity by activating AMP-activated protein kinase to regulate the expression of tight junction proteins [34]. Some carotenoids, such as astaxanthin and fucoxanthin, have been reported to ameliorate local and systemic inflammation in mice by enhancing the abundance of *Akkermansia* [38,39]. Similarly, mice fed the WPI-GA-ZD diet also showed the highest level of *Akkermansia*, and the abundance of this bacterium was inversely correlated with inflammation activity.

Subsequent PICRUSt analysis further confirmed that WPI-GA and WPI-GA-ZD played regulatory roles in different metabolic pathways. WPI-GA boosted the metabolic ability of carbohydrates, including "amino sugar and nucleotide sugar metabolism" and "fructose and mannose metabolism". According to a previous study, microbial dysbiosis could be improved by dietary intervention rich in non-digestible carbohydrates (enriching pathways for carbohydrate catabolism, such as “starch and sucrose metabolism” and “amino sugar and nucleotide sugar metabolism”) [40]. In the EZD group, the NOD-like receptor signaling pathway was significantly decreased after supplementing WPI-GA-ZD. It is well known that some NOD-like receptors are responsible for the maturation and secretion of pro-inflammatory cytokines, and several mutations in NOD-like receptor pathways are associated with inflammatory diseases [41]. The downregulation of the NOD-like receptor signaling pathway suggested the possible anti-inflammatory effects of WPI-GA-ZD.

## 5. Conclusions

In conclusion, taking protein-polysaccharide conjugates as the emulsifiers would not induce inflammation, whereas supplementation with WPI-GA and WPI-GA-ZD presented differential modulation of gut microbiota and played regulatory roles in different metabolic pathways. Notably, WPI-GA-ZD showed better effects in decreasing pro-inflammatory cytokines and endotoxins levels, increasing the SCFAs contents, improving the gut barrier and remodeling the gut microbiota structure. These findings confirmed the health -beneficial effects of ZD-enriched emulsion and provided a new basic theoretical basis for understanding the anti-inflammatory activity of carotenoid ester and protein-polysaccharide conjugates.

## Data Availability

The data presented in this study are available on request from the corresponding author.

## Supplementary Materials

The following supporting information can be downloaded at: https://www.mdpi.com/article/10.3390/foods11223670/s1, Figure S1. (A) CLSM images of the emulsions stabilized by WPI-GA Maillard conjugate (Nile Red was excited at 488 nm; Nile Blue was excited at 633 nm), (B) the enlarged view of emulsion droplets, (C) the particle size distribution of emulsion; Figure S2. Effects of WPI-GA and WPI-GA-ZD on the expression of serum pro-inflammatory cytokines and endotoxins. (A) TNF-α, (B) IL-6, (C) IFN-γ, (D) MCP-1, (E) LPS, (F) PGE2. Data are expressed as mean ± SD (n = 8). Different letters represent significant differences among different groups (*p* < 0.05); Figure S3. Effects of WPI-GA and WPI-GA-ZD on SCFAs contents. Data are expressed as mean ± SD (n = 8). Different letters represent significant differences among different groups (*p* < 0.05); Figure S4. Effects of WPI-GA and WPI-GA-ZD on the relative mRNA expression of recognition receptors and cytokines. (A) ZO-1, (B) occluding, (C) claudin-1, (D) iNOS, (E) IL-1β, (F) IFN-γ, (G) GPR41, (H) GPR43, (I) GPR109A. Data are expressed as mean ± SD (n = 6–8). Different letters represent significant differences among different groups (*p* < 0.05); Table S1. Primer sequence for qRT-PCR detection of colon tissue.
