# Supplementary material for: Zeaxanthin Dipalmitate-Enriched Emulsion Stabilized with Whey Protein Isolate-Gum Arabic Maillard Conjugate Improves Gut Microbiota and Inflammation of Colitis Mice
Source: Foods. 2022 Nov 16;11(22):3670. doi: 10.3390/foods11223670 (PMC9689712; doi:10.3390/foods11223670)
Supplement: Supplementary file 1 [file foods-11-03670-s001.zip › foods-2013379-supplementary.pdf]

foods MDPI

Supplementary Materials

Zeaxanthin Dipalmitate-Enriched Emulsion Stabilized with Whey Protein Isolate-Gum Arabic Maillard Conjugate Improves Gut Microbiota and Inflammation of Colitis Mice

Xuhui Kan [1], Wangting Zhou [1], Weiqi Xu [1], Zhuqing Dai [2], Yamei Yan [3], Jia Mi [3], Yi Sun [1], Xiaoxiong Zeng [1,*], Youlong Cao [3] and Lu Lu [3,*]

[1] College of Food Science and Technology, Nanjing Agricultural University, Nanjing 210095, China
[2] Institute of Agro-Product Processing, Jiangsu Academy of Agricultural Sciences, Nanjing 210014, China
[3] Institute of Wolfberry Engineering and Technology, Ningxia Academy of Agriculture and Forestry Sciences, Yinchuan 750004, China
* Correspondence: zengxx@njau.edu.cn (X.Z.); lulubing8901@163.com (L.L.) Tel.: +86-25-84396791 (X.Z.)

Characterization of emulsions stabilized by WPI-GA conjugates

The CLSM images of emulsions were presented in Figure S1. The green fluorescence represents the olive oil (A1 and B1), whereas the red fluorescence represented the protein-containing WPI-GA conjugates (A2 and B2). The overlap fluorescence image (A3 and B3) was obtained by exciting Nile Red and Nile Blue A. In the high magnification images (B1-B3), it was clearly shown that the green oil phase located in the interior, and WPI-GA conjugates tightly anchored to the surface of oil droplets, forming an oil-resistant barrier. The average particle size of emulsions was expressed by volume-weighted mean diameter *D*(4,3). The prepared emulsion showed a *D*(4,3) of 1.05 ± 0.05 μm and a ξ-potential value of -28.1 ± 0.5 mV.

Creaming index (CI) was used to evaluate the stability of emulsions stabilized by WPI-GA conjugates. Emulsions were stored in clear bottles with screw caps at 25 °C for 20 days. No clear phase separation was observed during storage. The excellent physical stability might be due to the improved emulsifying stability of Maillard conjugates, which decreased flocculation and coalescence by preventing droplet collisions.

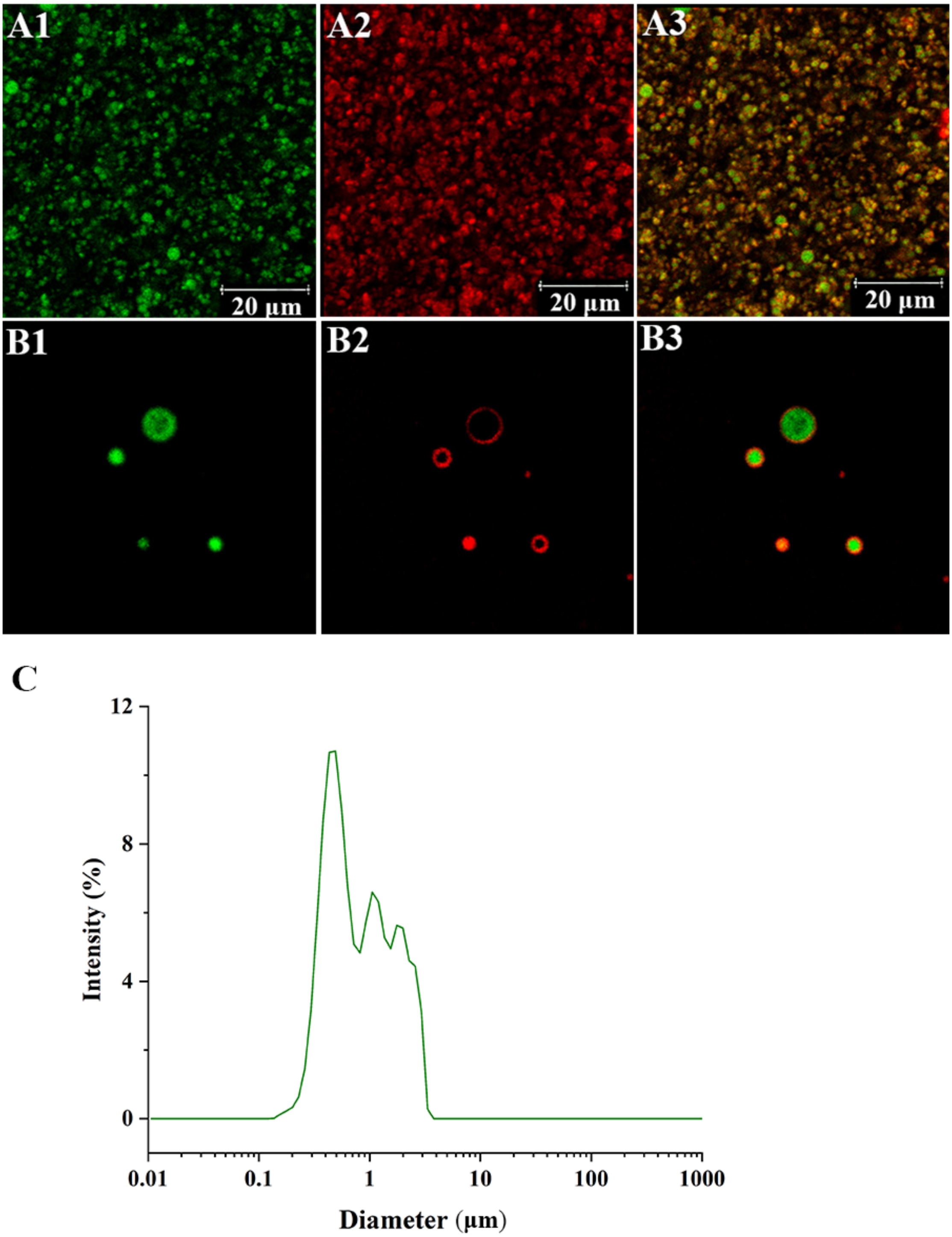

Figure S1. (**A1**–**A3**) CLSM images of the emulsions stabilized by WPI-GA Maillard conjugate (Nile Red was excited at 488 nm; Nile Blue was excited at 633 nm), (**B1**–**B3**) the enlarged view of emulsion droplets, (**C**) the particle size distribution of emulsion.

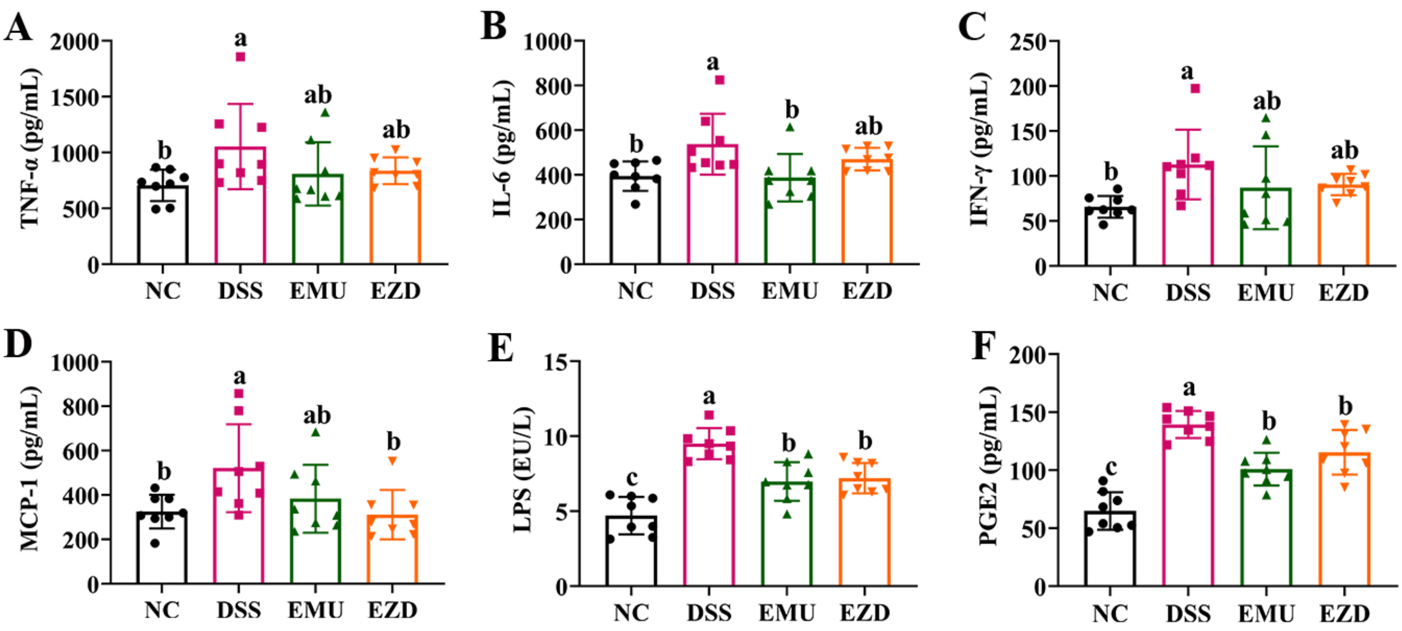

Figure S2. Effects of WPI-GA and WPI-GA-ZD on the expression of serum pro-inflammatory cytokines and endotoxins. (**A**) TNF-α, (**B**) IL-6, (**C**) IFN-γ, (**D**) MCP-1, (**E**) LPS, (**F**) PGE2. Data are expressed as mean ± SD (n = 8). Different letters represent significant differences among different groups ($p < 0.05$).

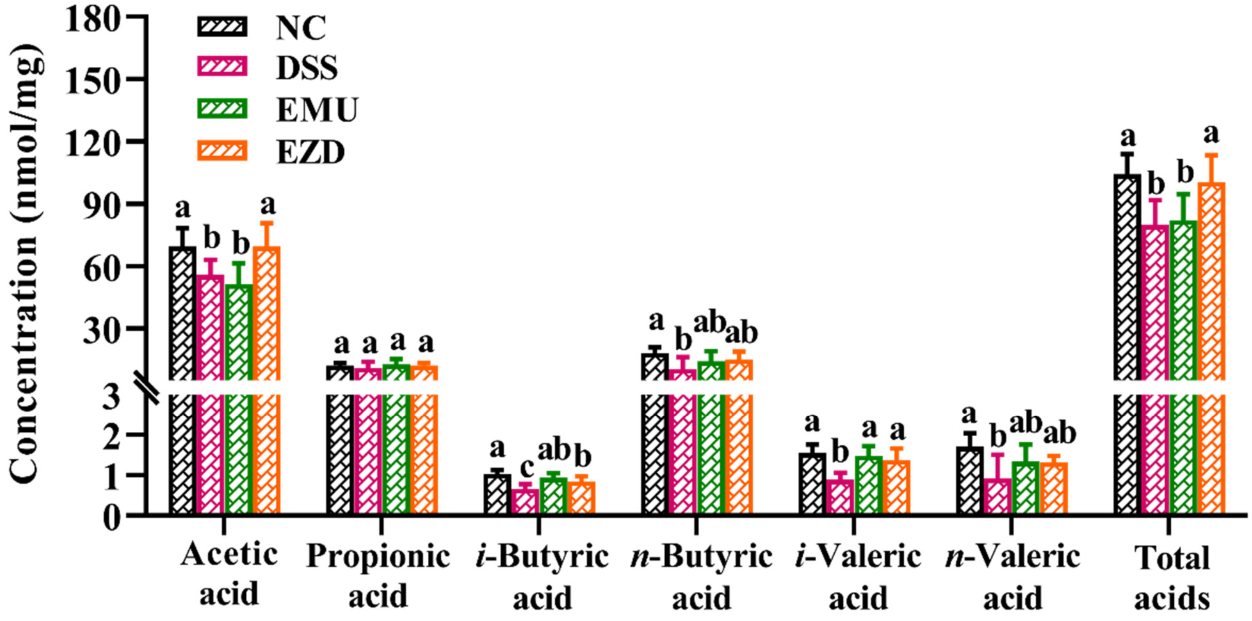

Figure S3. Effects of WPI-GA and WPI-GA-ZD on SCFAs contents. Data are expressed as mean ± SD (n = 8). Different letters represent significant differences among different groups ($p < 0.05$).

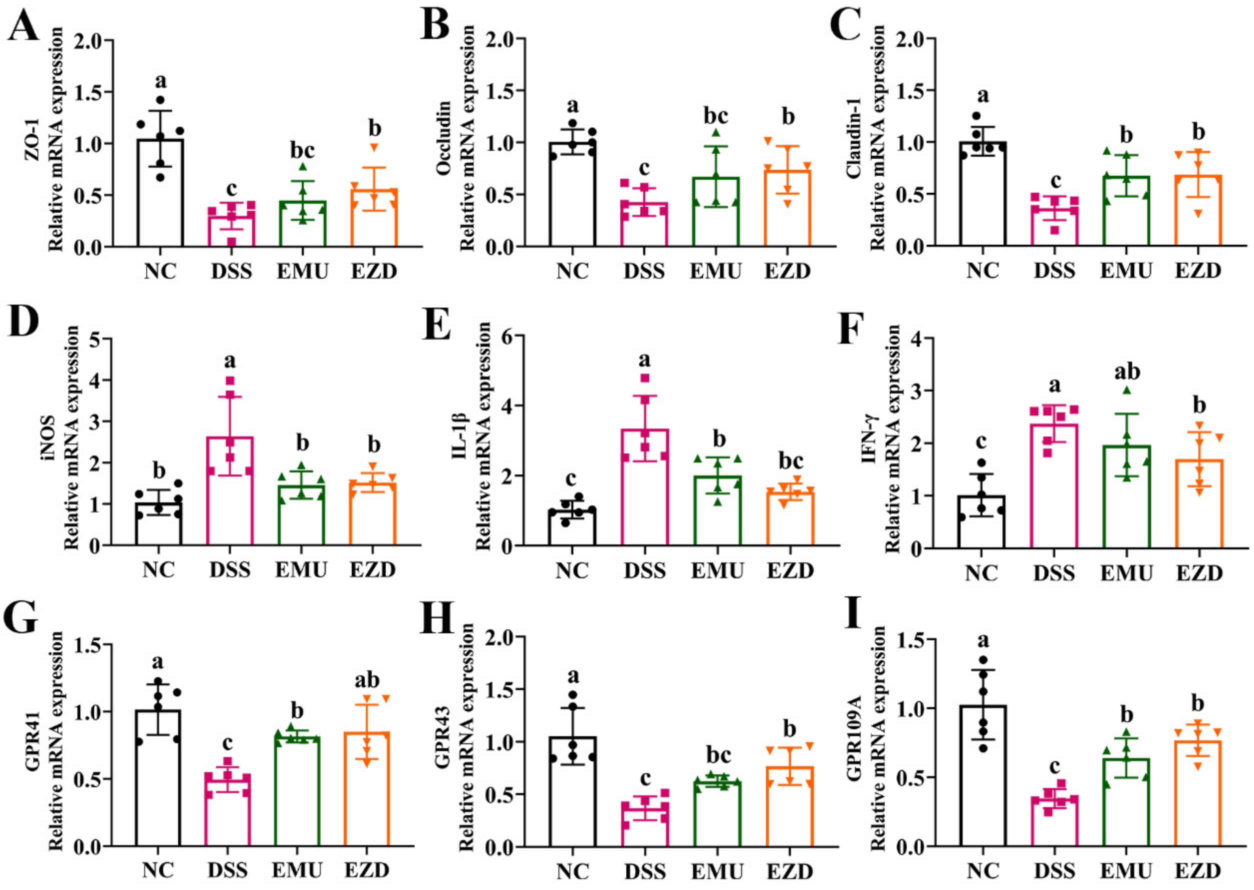

Figure S4. Effects of WPI-GA and WPI-GA-ZD on the relative mRNA expression of recognition receptors and cytokines. (**A**) ZO-1, (**B**) occluding, (**C**) claudin-1, (**D**) iNOS, (**E**) IL-1β, (**F**) IFN-γ, (**G**) GPR41, (**H**) GPR43, (**I**) GPR109A. Data are expressed as mean ± SD (n = 6-8). Different letters represent significant differences among different groups ($p < 0.05$).

Table S1. Primer sequence for qRT-PCR detection of colon tissue.

Target Gene	Primer Sequence (5´-3´)
IL-1β	FW: AGCTTCAAATCTCGCAGCAG<br>RV: TCTCCACAGCCACAATGAGT
IFN-γ	FW: AGCTCTTCCTCATGGCTGTT<br>RV: GGTCAACCAACCACAAGCAT
iNOS	FW: GGGCTGACCTGTTTCCTACT<br>RV: GGAGGTTGAGACCCAATGGA
ZO-1	FW: TGAGTGCGTTTCTCTCCCTT<br>RV: CCCTCTGTGTTCCTCATGGT
Occludin	FW: AGCACTTAACCTGCCTGGAT<br>RV: AGCCTGTGGAAGCAAGAGAT
Caludin-1	FW: AGCTGCCTGTTCCATGTACT<br>RV: CTCCCATTTGTCTGCTGCTC
GPR41	FW: CGACTAGAGATGGCTGTGGT<br>RV: AGAAGATGAGCAGTGTGGCT
GPR43	FW: TCCAGCCTGGCTTTCCAATA<br>RV: GCCTGCAGGAGACATTTCAG
GPR109A	FW: TCCAAGTCTCCAAAGGTGGT<br>RV: TGTTTCTCTCCAGCACTGAGTT
